# Quality of life in sarcopenia measured with the SarQoL®: impact of the use of different diagnosis definitions

**DOI:** 10.1007/s40520-017-0866-9

**Published:** 2017-12-01

**Authors:** Charlotte Beaudart, Médéa Locquet, Jean-Yves Reginster, Laura Delandsheere, Jean Petermans, Olivier Bruyère

**Affiliations:** 1 Research Unit in Public Health, Epidemiology and Health Economics (URSAPES), WHO Collaborating Centre for Public Health Aspects of Musculo-Skeletal Health and Aging, University of Liège, Avenue Hippocrate 13, CHU Bât B23, 4000 Liège, Belgium; 2 Geriatric Department, CHU Liège, Liège, Belgium

**Keywords:** Quality of life, Prevalence, Sarcopenia, SarQoL, Specific HRQoL questionnaire

## Abstract

**Background:**

The SarQoL® is a recently developed quality of life questionnaire specific to sarcopenia.

**Aim:**

To compare the quality of life (QoL) of subjects identified as sarcopenic with that of non-sarcopenic subjects when using six different operational definitions of sarcopenia.

**Methods:**

Participants of the SarcoPhAge study (Belgium) completed the SarQoL®. Among the six definitions used, two were based on low lean mass alone (Baumgartner, Delmonico), and four required both low muscle mass and decreased performance (Cruz-Jentoft, Studenski, Fielding, Morley). Physical assessments included measurements of muscle mass with dual energy X-ray absorptiometry, muscle strength with a handheld dynamometer and gait speed over a 4-m distance.

**Results:**

A total of 387 subjects completed the SarQoL®. Prevalence of sarcopenia varied widely across the different definitions. Using the SarQoL®, a lower QoL was found for sarcopenic subjects compared to non-sarcopenic subjects when using the definitions of Cruz-Jentoft (56.3 ± 13.4 vs 68.0 ± 15.2, *p* < 0.001), Studenski (51.1 ± 14.5 vs 68.2 ± 14.6, *p* < 0.001), Fielding (53.8 ± 12.0 vs 68.3 ± 15.1, *p* < 0.001), and Morley (53.3 ± 12.5 vs 67.1 ± 15.3, *p* < 0.001). No QoL difference between sarcopenic and non-sarcopenic subjects was found when using the definitions of Baumgartner or Delmonico, which were only based on the notion of decreased muscle mass.

**Discussion and conclusions:**

The SarQoL® was able to discriminate sarcopenic from non-sarcopenic subjects with regard to their QoL, regardless of the definition used for diagnosis as long as the definition includes an assessment of both muscle mass and muscle function. Poorer QoL, therefore, seems more related to muscle function than to muscle mass.

## Introduction

Sarcopenia, defined by a progressive loss of muscle mass and muscle function with advancing age, has been shown to be associated with several health consequences, such as a higher risk of functional decline, hospitalization, falls, fractures and death [1–4]. All of these consequences are likely to have a detrimental effect on health-related quality of life (HRQoL) [5–7]. Unfortunately, a very limited number of studies reported data for quality of life with sarcopenia through “Patient Reported Outcome” (PRO) tools. HRQoL assessments through PRO are increasingly important in research and clinical practice. The different purposes of PRO tools include obtaining accurate self-reported assessments of well-being and physical function and of the psychological and social implications of sarcopenic subjects but also increasing healthcare providers and regulatory agencies’ understanding of the needs and preoccupation of important segments of this population, such as elderly subjects suffering from sarcopenia.

For this purpose, a specific HRQoL questionnaire for sarcopenia, the SarQoL® [8, 9] was recently developed and validated. This self-administered questionnaire has been created with the objective of characterizing QoL in subjects with sarcopenia in research and in daily practice [10] but also to assess the relevance of therapeutic interventions in the field of sarcopenia by measuring their effectiveness in terms of changes in QoL. The SarQoL® has been validated in French, English and Romanian [9–12] and these three versions have been shown to be an understandable, valid and consistent questionnaire. During its validation analyses, the SarQoL® also showed its ability to discriminate sarcopenic subjects from non-sarcopenic subjects with regards to their HRQoL. However, one of the important public health issues regarding sarcopenia is the absence of an international consensus regarding its definition [13]. Indeed, since the very first definition of sarcopenia developed by Rosenberg in 1989 [14], which incorporated only the notion of decreased muscle mass, definitions have been expanded to incorporate the notion of decreased muscle function. Indeed, a higher decline in muscle strength than in muscle mass has been found in several epidemiological studies [15, 16], which highlighted the importance of this additional notion. Several operational definitions of sarcopenia have been developed and constitute attempts to establish a consensual clinically applicable definition of sarcopenia [3, 17–19]. Prevalence of sarcopenia can be dramatically different with regards to the definition used for the diagnosis but also the different criteria (threshold, tools, etc.) used across definitions [20–22]. Therefore, the discriminative power of the SarQoL® could be different according to the different existing definitions and criteria used for the diagnosis of sarcopenia.

Therefore, the purpose of this study was to compare the quality of life (QoL) of subjects identified as sarcopenic with that of non-sarcopenic subjects when using six different operational definitions of sarcopenia.

## Methods

### Population

The SarcoPhAge study (for Sarcopenia and Physical Impairment with advancing Age), which is an ongoing prospective study, was developed in Liège, Belgium, in June 2013 with the purpose of assessing the health and functional outcomes of sarcopenia. Subjects of the SarcoPhAge study are healthy subjects 65 years and older who were recruited in different departments of an outpatient clinic in Liège and through advertisements in the press. The methodological details of the study and baseline characteristics of the 534 subjects who were initially recruited as the SarcoPhAge population have been described in a previous study [23]. The present cross-sectional study is based on the population still participating in the SarcoPhAge study after 1 year of follow-up. All subjects were informed about the study objective and procedures. Informed written consent was given by all participants, and the research protocol and subsequent amendments were approved by the Ethics Committee of the University Teaching Hospital of Liège (number 2012/277).

### QoL assessment

The self-administered SarQoL® [8] is an HRQoL questionnaire specific for sarcopenia developed in 2014 (http://www.sarqol.org). Initially developed in French, the SarQoL® comprises 55 items translated into 22 questions. These items are organized in seven domains of dysfunction: physical and mental health, locomotion, body composition, functionality, activities of daily living, leisure activities and fears. A pre-test, which was performed on 20 sarcopenic subjects, indicated that the SarQoL® is comprehensible, is easy to complete independently, and can be completed in approximately 10–15 min [8]. The total possible score for the SarQoL® is 100 points. An individual score for each domain on 100 points can also be determined. The questionnaire has been shown to be understandable, valid, consistent, and reliable, and it can, therefore, be recommended for clinical and research purposes [9]. The questionnaire is now available in 13 different languages with another 20 language translations in progress.

### Diagnosis of sarcopenia

Through individual examinations, the following measures were collected by a clinical research assistant for all subjects:

- A measure of total muscle mass and appendicular lean mass using dual-energy X-ray absorptiometry [DXA, (Discovery A, Hologic)]. All whole-body scans were carried out by the same technician and the device was calibrated daily by scanning a spine phantom;
- A measure of handgrip muscle strength using a handheld dynamometer (Saehan Corporation, MSD Europe Bvba, Belgium) was calibrated at the beginning of the study for 10, 40 and 90 kg, that subjects had to squeeze as hard as possible three times with each hand (dominant and non-dominant). We used the highest result out of the six measurements recorded in our analysis [24];
- A measure of gait speed over a 4-m distance.

With these measurements, six operational definitions of sarcopenia were applied for the diagnosis (Table 1.). Two measurements were based on low lean mass alone (Baumgartner [25], Delmonico [26]), and four required both low muscle mass and decreased performance in a functional test (Cruz-Jentoft [3], Studenski [19], Fielding [17], Morley [18]).

**Table 1:** Operational definitions of sarcopenia applied

Criteria	Muscle mass	Muscle function		
		Muscle strength		Physical performance
Definitions based on low lean mass alone				
Baumgartner [25]	ALM/ht^2^ > 2 SD below young healthy mean	x		x
Delmonico [26]	ALM/ht^2^	x		x
	Men: ≤ 7.25 kg/m^2^			
	Women: ≤ 5.67 kg/m^2^			
Definitions requiring both low muscle mass and decreased muscle function				
Cruz-Jentoft [3] European Working Group on Sarcopenia in Older People (EWGSOP)	ALM/ht^2^	Grip strength	OR	Gait speed: < 0.8 m/s
	Men: ≤ 7.23 kg/m^2^	Men: < 30 kg		
	Women: ≤ 5.67 kg/m^2^	Women: < 20 kg	AND	
Fielding [17] International Working Group on Sarcopenia (IWGS)	ALM/ht^2^	x		Gait speed: < 1.0 m/s
	Men: ≤ 7.23 kg/m^2^			
	Women: ≤ 5.67 kg/m^2^			
Morley [18] Society of Sarcopenia, Cachexia and Wasting Disorders	ALM/ht^2^ > of 2 SD below the mean of healthy persons aged 20–30 years of the same ethnic group	x		Gait speed: ≤ 1.0 m/s or walking distance < 400 m during a 6-min walk
Studenski [19] Foundation of NIH Sarcopenia Project	ALM_BMI_	Grip strength		x
	Men: < 0.789	Men: < 26 kg		
			AND	Gait speed: ≤ 0.8 m/s
	Women: < 0.512	Women: < 16 kg		

*ALM/ht* ^*2*^ ratio of appendicular lean mass over height squared, *ALM*_*BMI*_ ratio of appendicular lean mass over body mass index, *SD* standard deviation

### Statistical analyses

The normality of variables was checked using the Shapiro–Wilk test. Continuous data are presented as the mean ± standard deviation (SD). Qualitative variables were reported as absolute and relative frequencies (%). Differences in QoL between sarcopenic subjects and non-sarcopenic subjects have been investigated through logistic regression. Age and sex were incorporated in all regression models as covariates. All analyses were performed using IMB SPPS Statistics 21.0. The results were considered statistically significant at the 5% critical level (*p* < 0.05).

## Results

**Fig. 1:**
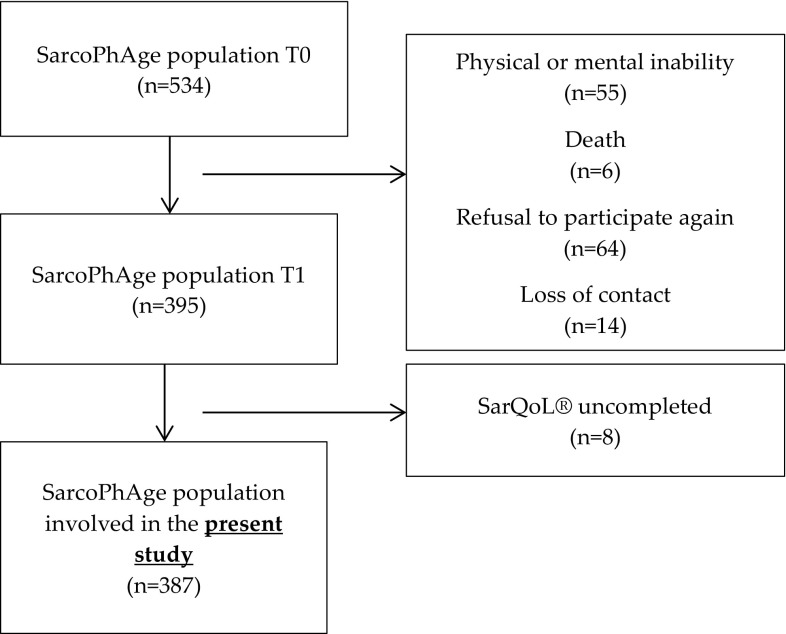
Involvement of participants in the SarcoPhAge Study

The SarQoL® questionnaire developed in 2014 has been cross-sectionally administered to all subjects of the SarcoPhAge study seen during their second year of follow-up. Among the 534 subjects recruited initially in 2013 for the SarcoPhAge study, several subjects (*n* = 139) were not interviewed the second year of follow-up in 2014 for various reasons: physical or mental inability (*n* = 55), death (*n* = 6), refusal to participate again (*n* = 64) or loss of contact (*n* = 14). A total of 395 participants were then identified as available for this ancillary analysis. Among the remaining 395 subjects, 387 presented sufficient clinical data to be included in the present study (Fig. 1).

**Fig. 2:**
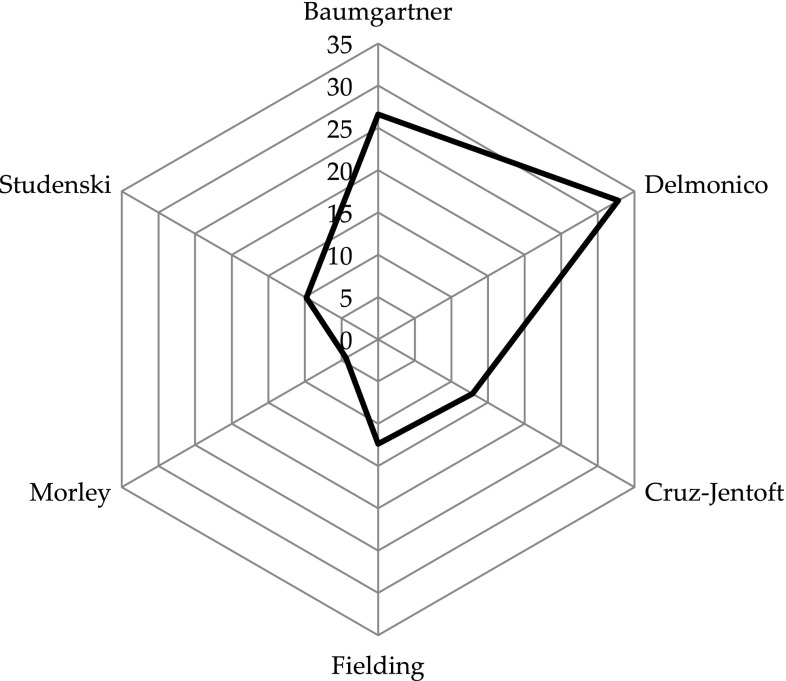
Prevalence of sarcopenia (%) according to diagnostic definitions

Characteristics of the population are described in Table 2. The mean age was of 74.02 ± 5.99 years, and 58.5% of the subjects were women. Mean Body Mass Index was 27.1 ± 4.96 kg/m^2^ and subjects presented globally a good cognitive status (mean MMSE of 28.7 ± 2.40 points out of 30 points) and a good nutritional status with 94.1% of the population having a good nutrition. Our population consumed a mean of 5.88 ± 3.43 drugs. The prevalence of sarcopenia varied widely across definitions, from 4.39% (*n* = 17) when sarcopenia was diagnosed according to Morley’s criteria [18] to 32.8% (*n* = 127) when sarcopenia was diagnosed according to Delmonico’s [26] criteria (Fig. 2).

**Table 2:** Characteristics of the population

Characteristics	Population (*n* = 387)
Age	74.02 ± 5.99
Sex	
Women	231 (58.5)
Anthropometric data	
Height	163.6 ± 9.57
Weight	72.9 ± 16.7
BMI	27.1 ± 4.96
Number of concomitant diseases	4.30 ± 2.46
Number of drugs	5.88 ± 3.43
Cognitive status (mini mental state examination)/30points	28.7 ± 2.40
Nutritionnal status (mini nutritional assessment)	
Good nutrition	364 (94.1)
At risk of malnutrition	22 (5.7)
Malnutrition	1 (0.2)
Depression (geriatric depression scale) (/8 points)	3.52 ± 3.27
Total lean mass (kg)	
Men	23.4 ± 3.78
Women	15.3 ± 2.56
Handgrip muscle strength (kg)	
Men	38.5 ± 8.92
Women	21.1 ± 7.15
Gait speed (m/s)	1.09 ± 0.29

Once adjusted for age and sex, a lower general QoL was found with the SarQoL® for sarcopenic subjects compared to non-sarcopenic subjects when using four definitions based on low muscle mass and low muscle function, including the definitions of Cruz-Jentoft [3] (56.3 ± 13.4 vs 68.0 ± 15.2, *p* < 0.001), Studenski [19] (51.1 ± 14.5 vs 68.2 ± 14.6, *p* < 0.001), Fielding [17] (53.8 ± 12.0 vs 68.3 ± 15.1, *p* < 0.001), and Morley [18] (53.3 ± 12.5 vs 67.1 ± 15.3, *p* < 0.001). No QoL difference between sarcopenic and non-sarcopenic subjects was found when using the definition of Baumgartner [25] (64.6 ± 15.8 vs 67.2 ± 15.3, *p* = 0.14) or Delmonico [26] (64.2 ± 15.2 vs 67.6 ± 15.5, *p* = 015), which were only based on aspects of decreased muscle mass (Table 3). The results were quite similar for individual domains of the SarQoL®, and the four definitions of sarcopenia based on low muscle mass and low muscle function distinguished the population of sarcopenic subjects from the population of non-sarcopenic subjects for all domains of HRQoL (D1: physical and mental health, D2: locomotion, D3: body composition, D4: functionality, D5: activities of daily living, D6: leisure activities and D7: fears). The only exception was for domain 7, which was not significantly lower for sarcopenic subjects compared to non-sarcopenic subjects when using the definition of Morley [18] (*p* = 0.13). However, for the two definitions based on muscle mass only, no difference in HRQoL has been found between sarcopenic and non-sarcopenic subjects for any of the domains, except for domains 5 and 7.

**Table 3:** SarQoL® scores across definitions of sarcopenia

	Definitions based on low lean mass alone		Definitions requiring both low muscle mass and decreased muscle function			
	Baumgartner [25]	Delmonico [26]	Cruz-Jentoft [3]	Fielding [17]	Morley [18]	Studenski [19]
D1. Physical and mental health						
Sarcopenic	61.9 ± 16.5	62.1 ± 15.7	56.3 ± 14.9	55.1 ± 13.0	55.2 ± 15.4	53.1 ± 14.9
Non-sarcopenic	65.6 ± 16.5	65.8 ± 16.9	65.8 ± 16.4	65.9 ± 16.6	65.0 ± 16.5	65.8 ± 16.3
*p* value	0.07	0.09	0.003	< 0.001	0.034	< 0.001
D2. Locomotion						
Sarcopenic	63.5 ± 22.1	62.9 ± 21.2	55.5 ± 19.7	48.5 ± 16.3	47.5 ± 17.1	45.8 ± 18.6
Non-sarcopenic	65.6 ± 16.5	63.8 ± 21.7	64.7 ± 21.6	65.6 ± 21.4	64.3 ± 21.4	65.5 ± 20.9
*p* value	0.85	0.91	0.042	< 0.001	0.007	< 0.001
D3. Body composition						
Sarcopenic	60.8 ± 16.1	60.8 ± 15.4	55.8 ± 14.8	54.3 ± 14.1	50.2 ± 13.4	56.2 ± 15.9
Non-sarcopenic	64.4 ± 16.8	64.7 ± 17.1	64.6 ± 16.6	64.7 ± 16.6	64.1 ± 16.5	64.2 ± 16.6
*p* value	0.08	0.08	0.004	0.001	0.003	0.025
D4. Functionality						
Sarcopenic	71.7 ± 16.8	71.3 ± 16.5	63.8 ± 17.3	60.8 ± 16.9	59.8 ± 17.4	56.7 ± 17.2
Non-sarcopenic	72.1 ± 15.7	72.3 ± 15.7	73.2 ± 15.4	73.5 ± 15.2	72.5 ± 15.7	73.6 ± 14.9
*p* value	0.98	0.98	0.004	< 0.001	0.008	< 0.001
D5. Activities of daily living						
Sarcopenic	61.4 ± 18.6	60.9 ± 18.2	49.8 ± 13.5	49.4 ± 14.0	48.5 ± 12.5	46.2 ± 18.8
Non-sarcopenic	66.6 ± 18.5	67.4 ± 18.5	67.5 ± 18.2	67.5 ± 18.1	66.0 ± 18.5	67.3 ± 17.4
*p* value	0.015	0.008	< 0.001	< 0.001	0.001	< 0.001
D6. Leisure activities						
Sarcopenic	54.3 ± 18.9	53.6 ± 18.4	48.6 ± 16.5	44.5 ± 14.6	40.6 ± 10.5	42.5 ± 13.5
Non-sarcopenic	55.2 ± 18.3	55.6 ± 18.4	55.9 ± 18.5	56.4 ± 18.4	55.6 ± 18.4	56.3 ± 18.4
*p* value	0.85	0.47	0.037	< 0.001	0.005	< 0.001
D7. Fears						
Sarcopenic	88.1 ± 11.4	87.9 ± 11.3	84.7 ± 12.4	83.3 ± 12.7	85.3 ± 11.9	81.6 ± 13.8
Non-sarcopenic	90.4 ± 10.3	90.7 ± 10.2	90.6 ± 10.1	90.7 ± 9.97	90.0 ± 10.5	90.7 ± 9.82
*p* value	0.07	0.04	0.002	< 0.001	0.13	< 0.001
Total scores of the SarQoL						
Sarcopenic	64.6 ± 15.8	64.2 ± 15.2	56.3 ± 13.4	53.8 ± 12.0	53.3 ± 12.5	51.1 ± 14.5
Non-sarcopenic	67.2 ± 15.3	67.6 ± 15.5	68.0 ± 15.2	68.3 ± 15.1	67.1 ± 15.3	68.2 ± 14.6
*p* value	0.19	0.15	< 0.001	< 0.001	0.002	< 0.001

*All *p* values are adjusted on age and sex

## Discussion

The co-existence of different diagnostic criteria for sarcopenia represents a major public health issue. Indeed, several studies have shown considerable variation in the prevalence of sarcopenia when using a different definition of sarcopenia [20, 27, 28]. The present study also highlights the variation in the prevalence of sarcopenia when using six operational definitions of sarcopenia. This prevalence varied in our sample from 4.39 to 32.8%. Obtaining a prevalence that is dependent on the diagnostic criteria used for the diagnosis could lead to important consequences from a public point of view. For example, an over- or underestimation of the prevalence of sarcopenia could impact therapeutic or preventive interventions by increasing the risk of giving unnecessary treatment to a false positive subject (i.e., without sarcopenia) and depriving a false negative patient (i.e., with sarcopenia) of effective treatment [29, 30].

In regards of QoL, the SarQoL® showed its ability to discriminate sarcopenic subjects with regards to their QoL whatever the operational definition of sarcopenia that was used as long as the definition included an assessment of both muscle mass and muscle function. Indeed, when using oldest definitions of sarcopenia, which focused only on decreased muscle mass, the sarcopenic subjects did not show a decreased QoL compared to non-sarcopenic subjects. Poorer QoL, therefore, seems to be more related to muscle function than to muscle mass. Surprisingly, even domain 3, including questions related to body composition, did not differ between groups for the two muscle-mass only definitions. It should be noted, nevertheless, that two definitions based solely on muscle mass were the two definitions associated with the highest prevalence of sarcopenia. It could then be hypothesized that these definitions did not identify subjects with worse musculoskeletal health. By definition, the SarQoL® is a specific HRQoL for sarcopenia and muscle impairments. All of the questions present in this questionnaire are related to muscle health. It is, therefore, not surprising to obtain the highest difference in QoL between sarcopenic and non-sarcopenic subjects when sarcopenia is diagnosed by definitions that identify subjects with worse musculoskeletal health (i.e., not only decreased muscle mass but also decreased muscle function). This aspect was already shown in the initial validation of the SarQoL® with results indicating that QoL scores for severe sarcopenic subjects were even lower than the ones obtained by sarcopenic subjects, which indicated that the questionnaire was able to capture the severity of sarcopenia [9]. During the English validation of the SarQoL®, which was conducted with 235 participants from the Hertfordshire study [31, 32] in the UK, the results were quite similar. Indeed, a lower QoL was found for sarcopenic subjects (61.9 ± 16.5) compared to non-sarcopenic subjects (71.3 ± 12.8; *p* = 0.01) but only 14 subjects were diagnosed sarcopenic (prevalence of 5.96%). To perform validation analyses on a higher number of participants, modified cut-offs have been applied to diagnose a larger group of subjects, not with sarcopenia but with “a lower global muscle function”. These new-cut-offs, which are less restrictive than the original ones, led to a prevalence of 39.6%, which seems closer to the prevalence found with the criteria of Baumgartner and Delmonico in our study. No difference in QoL between subjects with a lower global muscle function and subjects with a normal muscle function has been observed. These results additionally indicate that the SarQoL® should be used in a population with the most affected muscle mass and function.

It has to be pointed that, despite the strengths of this study, which include a large sample of subjects, the ability to apply six different definitions of sarcopenia to our sample and the originality of data collected, this study could be prone to selection bias. Indeed, by selecting voluntary subjects, our population may not be fully representative of subjects suffering from sarcopenia and is, therefore, limited in its external validity. QoL of sarcopenic subjects should be worse than determined in this study because voluntary subjects who participated in our study were still independent and able to walk and presented sufficient cognitive status. Moreover, it should be acknowledged that some confounding factors have not been taken into account. Indeed, we do not have information about specific diseases or social support of participants that could impact sarcopenia and quality of life. Additionally, the transversal design of our study leads only to an analysis of static sarcopenia and not to an analysis of dynamic sarcopenia.

The SarQoL® can discriminate sarcopenic from non-sarcopenic subjects in regard to their QoL regardless of the definition used for the diagnosis as long as the definition includes an assessment of both muscle mass and muscle function. Poorer QoL, therefore, seems to be more related to muscle function than to muscle mass. These results are important for potential future treatments of sarcopenia, which aim not only to treat sarcopenia but also to improve the HRQoL of subjects suffering from sarcopenia.
